# Mid-Term Clinical Outcomes of the Low-Profile Ankura™ Stent Graft System for Endovascular Aneurysm Repair

**DOI:** 10.3390/jcm15093231

**Published:** 2026-04-23

**Authors:** Fatma Akca Ozsar, Bekir Bogachan Akkaya, Mehmet Cahit Saricaoglu, Onur Buyukcakir, Evren Ozcinar, Hakki Zafer Iscan, Levent Yazicioglu

**Affiliations:** 1 Department of Cardiovascular Surgery, Kirikkale High Specialization Hospital, 71100 Kirikkale, Turkey; akcaafatma@gmail.com; 2 Department of Cardiovascular Surgery, Ankara Bilkent City Hospital, 06800 Ankara, Turkey; 3 Department of Cardiovascular Surgery, Ankara University, 06100 Ankara, Turkey

**Keywords:** abdominal aortic aneurysm, EVAR, Ankura™ stent graft, endoleak, reintervention, mid-term safety

## Abstract

**Background:** To evaluate the real-world safety and mid-term clinical performance of the Ankura™ AAA Stent Graft System in patients undergoing endovascular aneurysm repair (EVAR). **Materials and Methods:** This prospective, multicenter PMCF study analyzed 100 patients with abdominal aortic aneurysms (AAAs). Patients were monitored for a mean duration of 2.26 years. Primary endpoints included 30-day major adverse events and 24-month treatment success. Statistical evaluation of risk factors for reintervention was performed using univariate logistic regression. **Results:** The study cohort was predominantly male (97%), with a mean age of 72.01 years. Hypertension (90%) and smoking (89%) were the most prevalent comorbidities. Regarding the primary endpoints, the 30-day MAE rate was 2%. During the overall follow-up (mean 2.26 years), the primary patency rate was 97%, demonstrating high structural integrity and sustained patency. However, the overall freedom from reintervention rate was 74%, corresponding to a 26% reintervention requirement and a 27% incidence of endoleak. Reinterventions were almost exclusively driven by these post-procedural morphological complications; specifically, 26 of the 27 patients with endoleaks required a secondary procedure. No preoperative clinical or anatomical parameters were identified as significant independent predictors of reintervention in the univariate analysis (*p* > 0.05). The overall mortality rate was 12%, with 0% aneurysm-related mortality. **Conclusions:** Mid-term success and reintervention after EVAR with the Ankura™ system are primarily driven by postoperative morphological complications, such as endoleaks, rather than baseline patient risk profiles. These findings underscore the critical importance of rigorous, lifelong radiological surveillance regardless of preoperative anatomical challenges.

## 1. Introduction

The management of infrarenal abdominal aortic aneurysms (AAAs) has been fundamentally transformed by endovascular aneurysm repair (EVAR), which is now recommended as the first-line treatment for patients with anatomically suitable characteristics. Due to its less invasive nature, EVAR offers significantly lower perioperative morbidity and improved early outcomes compared to open surgical repair (OSR) [1,2,3,4,5]. However, landmark randomized controlled trials, such as EVAR-1 and OVER, have demonstrated that long-term survival outcomes between EVAR and OSR remain similar [6,7]. Furthermore, nearly 30% of patients undergoing EVAR require reintervention within 10 years of the index procedure [8,9], necessitating obligatory lifelong surveillance. Consequently, achieving sustained graft stabilization in the face of complex anatomical constraints—which often lead to endoleaks and reinterventions—remains a critical clinical challenge.

To overcome these challenging access anatomies, which are frequently encountered in female patients and those with narrow or calcified iliac vessels, modern endovascular repair increasingly utilizes low-profile delivery systems [10,11]. The low-profile Ankura™ Stent Graft System(Lifetech Scientific, Shenzhen, China) has emerged as a reliable device designed to navigate these progressively smaller vessel accesses, potentially reducing procedural morbidity. However, while other contemporary low-profile systems have been extensively studied, there remains a notable evidence gap regarding the mid-to-long-term performance of the Ankura™ system in a modern, unselected patient population. Much of the existing literature on this specific device is either dated or limited to narrow cohorts, necessitating updated “real-world” data to understand its efficacy in preventing late endoleak formation and subsequent reinterventions.

In order to evaluate the mid-to-long-term safety and performance of the Ankura™ portfolio (including AAA, Cuff, and AUI stent grafts) in a real-world setting, this PMCF study was planned in accordance with Medical Device Regulation (MDR) requirements. This study aims to assess mid-term clinical performance, with a particular emphasis on postoperative clinical outcomes and the need for secondary interventions.

## 2. Materials and Methods

### 2.1. Study Design

This multicenter, prospective, single-arm, open-label, post-market clinical follow-up (PMCF) study was designed to evaluate the real-world safety and performance of the Ankura™ AAA Stent Graft Systems (Lifetech Scientific, Shenzhen, China). The study protocol was conducted in accordance with the principles of the Declaration of Helsinki and received formal approval from the Institutional Review Board (Protocol No: 2023000471-1(2023/471); Approval No: I08-546-23; Date: 14 September 2023).

Prior to enrollment, all participants were provided with detailed information regarding the study objectives and longitudinal monitoring, and written informed consent was obtained from each patient.

**Figure 1:**
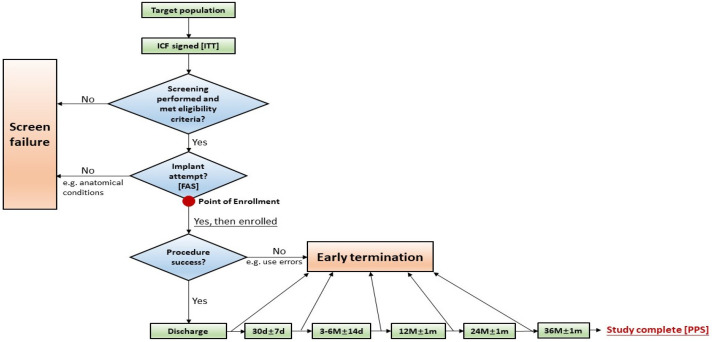
CONSORT-style flow diagram of the clinical study design and follow-up schedule. ITT: Intention-to-treat; FAS: Full Analysis Set; PPS: Per-Protocol Set.

The systematic progression of subjects from recruitment through the final 36-month follow-up, including the definition of analysis sets such as Intention-to-Treat (ITT), Full Analysis Set (FAS), and Per-Protocol Set (PPS), is detailed in the study flow algorithm (Figure 1). Patients were consecutively enrolled between February 2023 and February 2024 across two tertiary clinical centers. Although the primary study protocol involves a longitudinal monitoring period of 36 months to assess long-term durability, the present study represents an interim analysis reflecting mid-term clinical outcomes with a mean follow-up duration of 2.26 ± 0.53 years. Long-term data will be updated and reported as the 36-month follow-up for the entire cohort is completed.

#### 2.1.1. Standardized Follow-Up and Imaging Protocol

A comprehensive, standardized data collection and follow-up protocol was implemented. Baseline assessments included comprehensive physical examinations (incorporating NYHA and ASA classifications), laboratory tests, and fine-cut computed tomography angiography (CTA, slice thickness ≤ 2.5 mm) to acquire precise preoperative anatomical measurements for exact graft sizing. Post-procedure, patients were systematically evaluated prior to discharge, and subsequently at 30 days, 3–6 months, 12 months, 24 months, and 36 months.

To meticulously monitor graft performance, structural integrity, and aneurysm sac dynamics (including endoleak detection), fine-cut CTA imaging alongside clinical and laboratory assessments was strictly mandated at the 3–6 month, 12-month, and 24-month intervals. The 30-day and 36-month follow-ups were designed primarily as remote or telephone visits focusing on adverse event (AE) reviews and medication adherence, with on-site clinical evaluations or supplemental imaging performed solely at the investigator’s discretion. Any patient returning between scheduled intervals was evaluated as an unscheduled visit. All imaging data were reviewed to assess anatomical changes over the longitudinal follow-up period.

#### 2.1.2. Inclusion and Exclusion Criteria

Patients were screened for eligibility based on a diagnosis of abdominal aortic aneurysm (AAA) requiring elective endovascular repair. The study population was strictly defined by anatomical and clinical parameters to ensure compliance with the Ankura™ AAA and Aorto-Uni-Iliac Stent Graft Systems Instructions for Use (IFU).

##### Inclusion Criteria

To be eligible for enrollment, patients had to meet the following anatomical requirements:

- Proximal Aortic Neck: A non-aneurysmal length of ≥15 mm, a diameter between 18 and 32 mm, and an angulation ≤ 60°.
- Iliac Anatomy: A distal anchorage zone of ≥15 mm with adequate iliac/femoral access vessels. Specific distal iliac diameters were required: 8–22 mm for the bifurcated/cuff systems and 10–16 mm for the AUI system.
- Consent: Patients were required to provide written informed consent and demonstrate a willingness to comply with the 3-year longitudinal follow-up protocol.

##### Exclusion Criteria

Patients were excluded based on the following contraindications:

- Demographics and Life Expectancy: Age < 18 or >85 years, life expectancy < 1 year, or pregnancy.
- Anatomical Constraints: Inadequate proximal neck dimensions (length < 15 mm, diameter < 18 mm or >32 mm), extreme neck angulation (>60°), presence of thrombus in the aneurysm neck, or concomitant thoracic/thoraco-abdominal aneurysms.
- Clinical Comorbidities: Acute systemic infection, uncorrectable coagulopathy, severe renal insufficiency (precluding contrast use), or hereditary connective tissue disorders (e.g., Marfan or Ehlers-Danlos syndromes).
- Physiological Limitations: Obesity (>150 kg) prevents accurate fluoroscopy, or severe systemic conditions such as NYHA Class III/IV heart failure, recent myocardial infarction, or respiratory failure.
- Technical Considerations: Dependence on the inferior mesenteric artery for mesenteric perfusion or the presence of accessory renal arteries originating from the aneurysm.

Final eligibility was subject to the investigator’s clinical judgment regarding the patient’s overall suitability for endovascular intervention.

#### 2.1.3. Device Description

The Ankura™ AAA Stent Graft System is a modular, bifurcated endoprosthesis featuring a sutureless ePTFE film supported by a nitinol mesh. The current low-profile generation (18–23 Fr) incorporates iterative design enhancements, including a modified barb design for improved proximal fixation and enhanced catheter flexibility for navigating tortuous anatomy. To ensure connection integrity, recent optimizations include a distal “pocket” on the main body’s short branch and a bare spring on the proximal cuff, facilitating stable overlapping. All components are delivered via the Ankura™ Delivery System using. As a PMCF study, all devices are CE-marked, used within their approved indications, and tracked via serial numbers in the Case Report Forms (CRF).

#### 2.1.4. Operative Technique

All endovascular procedures were performed in a standard hybrid operating room by a dedicated vascular surgery team. All procedures were conducted under general anesthesia. Vascular access was achieved via a standard surgical cut-down of the common femoral arteries. Systemic heparinization was administered to maintain an activated clotting time (ACT) > 250 s. In accordance with routine endovascular aneurysm repair (EVAR) protocols, the Ankura™ main body was deployed first, followed sequentially by the placement of the limb extensions under fluoroscopic guidance. Following deployment, completion angiography was routinely performed to confirm stent-graft patency, correct positioning, and the absence of immediate endoleaks.

#### 2.1.5. Outcome Measures

##### Primary Endpoints

The primary safety endpoint was defined as the 30-day incidence of major adverse events (MAE), comprising all-cause mortality, myocardial infarction or cardiac failure, stroke and other systemic neurological damage, intestinal ischemia, and acute renal insufficiency. The primary effectiveness endpoint was the 24-month treatment success rate, evaluated as a composite outcome including technical success (defined as the accurate placement and successful deployment of the endoprosthesis), absence of aneurysm rupture or conversion to open surgery, stable sac diameter (expansion ≤ 5 mm), and freedom from device-related complications such as stent migration (>10 mm), graft material rupture, and thrombosis. Additionally, freedom from re-intervention specifically addressed Type I and Type III endoleaks within this period.

##### Secondary Endpoints

Secondary endpoints evaluated through the 36-month follow-up period include all-cause and aneurysm-related mortality, and the cumulative incidence of procedure-related serious adverse events (SAE). SAEs were categorized based on clinical presentation, including arterial damage or perforation, arteriovenous fistula, pseudoaneurysm, and significant bleeding or hematoma formation. Furthermore, systemic complications such as pulmonary issues, erectile dysfunction, infection, and wound-related problems were documented.

Longitudinal performance is monitored at 3–6, 12, and 24 months via the incidence of endoleaks (Types I–IV), and stent-related structural integrity, including potential migration, fracture, erosion, or thrombosis. Post-operative CTA scans were evaluated locally by the surgical team and an independent cardiovascular radiologist. Morphological assessments focused on maximum sac diameter (outer-to-outer, orthogonal to the centerline), endoleak classification, and device integrity, with any observer discrepancies resolved by consensus. The technical performance of the delivery system, specifically focusing on insertion or release difficulties and the accuracy of placement, was recorded as an immediate procedural success metric. Finally, any instances of late aneurysm enlargement or vessel rupture were analyzed as secondary indicators of mid-to-long-term graft durability.

### 2.2. Statistical Analysis

Statistical analysis was performed using IBM SPSS Statistics software (version 31.0; IBM Corp., Armonk, NY, USA). Continuous variables are presented as mean ± standard error (SE) and median (minimum–maximum), while categorical variables are reported as frequencies (*n*) and percentages (%). The normality of continuous variables was assessed using skewness and kurtosis coefficients; values within the range of ±1.96 were considered indicative of a normal distribution. Additionally, normality was confirmed using the Shapiro–Wilk test. For the comparison of continuous variables between two groups, the independent samples *t*-test was employed for normally distributed data. Categorical variables were compared using the Chi-square (x^2^) test, and Fisher’s exact test was applied when expected cell counts were less than 5.

The primary outcome variables were defined as the presence of endoleak, reintervention requirement, and mortality. These variables were coded as binary/dichotomous outcomes (0 = No/Alive, 1 = Yes/Exitus). To identify potential risk factors affecting the requirement for reintervention, univariate logistic regression analysis was performed. The results are presented as the regression coefficient (B), standard error (SE), odds ratio (OR = Exp(B)), and 95% confidence interval (CI). Statistical significance was defined as a *p*-value < 0.05.

## 3. Results

### 3.1. Patient Flow and Characteristics

A total of 100 patients who underwent Endovascular Abdominal Aortic Aneurysm Repair (EVAR) were included in the final analysis. The primary technical success rate, defined as the accurate and successful deployment of the endoprosthesis without intraoperative conversion to open surgery, was achieved in 100% of the cases. The study population was predominantly male (97%, *n* = 97) with a mean age of 72.01 ± 5.32 years. The majority of patients (71%, *n* = 71) were in the ≥70 years age group. Key preoperative comorbidities included hypertension in 90% (*n* = 90), smoking in 89% (*n* = 89), and dyslipidemia in 51% (*n* = 51) of the cohort. Functional and clinical assessments revealed that 51% (*n* = 51) were classified as NYHA II, and 67% (*n* = 67) had an ASA physical status classification of II (Table 1).

### 3.2. Primary Safety and Effectiveness Endpoints

The primary safety endpoint, defined as the 30-day incidence of major adverse events (MAE), occurred in only 2% (*n* = 2) of the cohort. One patient experienced an acute myocardial infarction managed successfully with percutaneous coronary intervention, while the second developed acute kidney injury that resolved fully with conservative treatment. Over a mean follow-up of 2.26 years, the device demonstrated excellent durability. Although three patients developed non-flow-limiting partial limb thrombosis, these were successfully managed with conservative medical therapy, yielding a 100% secondary patency rate.

**Figure 2:**
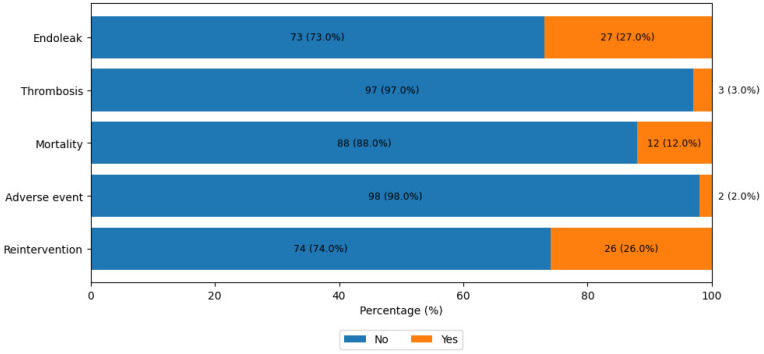
Incidence and distribution of endoleak, thrombosis, mortality, adverse events, and reintervention requirements in the patient cohort.

Regarding aneurysm sac dynamics over the follow-up period, sac regression (≥5 mm decrease) was achieved in 23% (*n* = 23) of the cohort, all of whom were free from endoleaks. Sac stabilization (change within ±5 mm) was observed in the majority of patients (74%, *n* = 74). This stabilized group comprised 50 patients without endoleaks, one patient with a conservatively managed Type II endoleak, and 23 patients with Type Ib endoleaks, whose primary aneurysm sacs remained stable despite the distal iliac progression that eventually necessitated extension. Conversely, sac expansion (>5 mm) occurred in only 3% (*n* = 3) of the patients. This expansion was strictly associated with early-onset Type Ia endoleaks, prompting immediate reintervention. Consequently, the overall freedom from reintervention rate was 74%, which was inversely driven by 26 patients requiring secondary procedures exclusively for endoleak management (Figure 2).

### 3.3. Mid-Term Clinical Outcomes and Factors Associated with Reintervention

During the follow-up period (mean duration 2.26 ± 0.53 years), the overall incidence of endoleak was 27% (*n* = 27), and the reintervention requirement was documented in 26% (*n* = 26) of the patients. In this cohort, the necessity for secondary reintervention was almost exclusively driven by postoperative morphological complications. Specifically, 26 out of the 27 patients who developed an endoleak required a secondary procedure to ensure graft stability, with the specific timings of these prompt reinterventions detailed in Table 2.

Detailed analysis of these reinterventions revealed that the vast majority (*n* = 23) were necessitated by Type Ib endoleaks. Notably, these Type Ib endoleaks predominantly occurred during late follow-ups secondary to progressive aneurysmal dilatation of the iliac arteries. These cases were successfully treated with the deployment of distal extensions. The remaining 3 cases involved Type Ia endoleaks, which were effectively managed with the placement of proximal cuffs to restore and secure the proximal seal. The single remaining patient from the overall endoleak cohort presented with a Type II endoleak, which was managed conservatively with close surveillance without the need for reintervention, given the absence of significant sac expansion.

The overall all-cause mortality rate was 12% (*n* = 12); importantly, the aneurysm-related mortality (ARM) rate was 0%. Two of these deaths occurred during the initial 12-month follow-up period due to cardiac and malignancy-related complications. The remaining 10 late mortalities were also entirely driven by non-aneurysmal causes, specifically including cardiac events (*n* = 4), respiratory failure (*n* = 3), underlying malignancies (*n* = 2), and a motor vehicle accident (*n* = 1). Detailed distributions of these clinical outcomes are graphically represented in Figure 2.

The follow-up study concentrated on determining possible relationships between baseline preoperative characteristics and the need for secondary interventions. In this cohort, no statistically significant differences were observed between patients with and without reintervention regarding demographic variables such as age, gender, or smoking history (Table 1). Furthermore, systemic comorbidities, including diabetes mellitus, coronary artery disease, and peripheral artery disease, did not appear to be significantly associated with an increased likelihood of reintervention within our study population (Table 1). Similarly, preoperative hemodynamic, laboratory, and anatomical measurements—including baseline aneurysm diameter and proximal neck length—were comparable between the two groups (Table 3).

To further evaluate potential independent risk factors, a univariate logistic regression analysis was performed. This analysis demonstrated that no preoperative demographic, clinical, or anatomical parameter including age ≥ 70 (OR: 1.503; 95% CI: 0.533–4.239, *p* = 0.441), hypertension (OR: 3.462; 95% CI: 0.417–28.748, *p* = 0.250), or aneurysm diameter (OR: 1.009; 95% CI: 0.968–1.053, *p* = 0.671) was a significant independent predictor of reintervention (Table 4). Collectively, these findings support that the necessity for secondary procedures in this patient group was governed by post-procedural morphological developments, such as endoleak detection, rather than identifiable preoperative risk profiles.

## 4. Discussion

The contemporary management of abdominal aortic aneurysms (AAAs) has shifted toward endovascular aneurysm repair (EVAR) as the primary treatment modality, largely due to its significantly reduced perioperative morbidity and mortality compared to open surgical repair [4,5]. In our clinical follow-up study involving 100 patients, the demographic profile—predominantly elderly males (97%) with a high prevalence of hypertension (90%) and a significant history of smoking (89%) strongly aligns with established epidemiological data. These baseline characteristics validate that our cohort represents a typical “real-world” EVAR population, providing a reliable foundation for analyzing mid-term outcomes and secondary interventions [2,5].

The selection of the Ankura™ stent graft in our cohort is particularly justified by its design catalog specifications, notably its low-profile delivery system (ranging from 18 Fr to 23 Fr) and its high-quality hydrophilic coating. These design features offer a substantial clinical advantage by facilitating smoother navigation and deployment in patients presenting with challenging, narrow, or tortuous access vessel anatomies. By mitigating access-related difficulties, this low-profile system contributes to a safer procedural profile.

Specifically, we observed a 27% incidence of endoleaks, which exclusively drove our 26% reintervention rate (Table 2). While this reintervention rate is notably higher than the 10–20% typically reported in some contemporary EVAR series at 2 years, it must be contextualized. First, this rate was likely influenced by ascertainment bias due to our rigorous, protocol-driven CTA surveillance, which effectively captured even asymptomatic morphological changes. Second, when compared to landmark trials, our findings parallel the long-term realities of EVAR; for instance, the EVAR-1 trial reported a reintervention rate of approximately 20% at 4 years, and similar persistent risks were noted in the OVER trial [3,4,6,7]. In our cohort, Type Ib endoleaks accounted for the vast majority of these secondary procedures (23%). Rather than indicating inherent device failure, these distal leaks primarily resulted from the natural, progressive dilatation of the iliac arteries over time and the complex baseline iliac anatomies of the treated patients (such as high tortuosity and severe calcification), though we must acknowledge that suboptimal iliac sizing (oversizing) at the index procedure might have also contributed to this phenomenon. This highlights the challenge of ongoing iliac disease post-EVAR, pointing to the need for proactive distal sizing or earlier use of iliac branch devices (IBDs) in patients with baseline iliac ectasia. Ultimately, despite the need for these distal extensions, the core Ankura™ graft achieved a 97% patency rate with zero aneurysm-related mortality.

Our univariate logistic regression identified no significant demographic, systemic, or morphological predictors for reintervention (*p* > 0.05). However, we must explicitly acknowledge the limited statistical power of this analysis as a constraint. With a cohort of 100 patients and only 26 reintervention events, the study is underpowered to detect subtle predictors via multivariable modeling. Therefore, these null results should not be over-interpreted as true negatives, and baseline anatomical severity may still play a crucial role in larger populations. Classical risk factors for Type Ia endoleak, such as proximal neck length and angulation, did not reach statistical significance, likely due to our strict adherence to the manufacturer’s Instructions for Use (IFU; neck length ≥ 15 mm). Although patients requiring reintervention had numerically shorter proximal necks (18.85 ± 3.57 mm vs. 20.97 ± 6.73 mm), this trend was not significant. While complex anatomy historically limits EVAR durability, our very low rate of Type Ia endoleaks (*n* = 3) suggests that the Ankura™ system’s enhanced proximal fixation effectively mitigates these proximal anatomical challenges. Furthermore, our technical success aligns with the principles demonstrated in the LUCY trial; although the LUCY trial specifically investigated the Ovation device rather than the Ankura system, it validated the broader concept that low-profile delivery systems provide a distinct advantage in navigating difficult vascular access [10,11,12,13].

Finally, we documented an overall mortality rate of 12% over the mid-term follow-up period, with an aneurysm-related mortality (ARM) rate of precisely 0%. While early survival post-EVAR is typically excellent (as reflected by our 2% 30-day MAE rate), a synthesis of our mortality data reveals a clear temporal division. Early mortality (within the first 12 months) accounted for only two deaths, driven by pre-existing cardiac and malignancy-related complications. Conversely, the 10 late mortalities were entirely attributable to non-aneurysmal systemic issues, specifically cardiovascular events, respiratory failure, and malignancies. This trend reflects the natural attrition in this high-risk cardiovascular population [14,15]. These outcomes, coupled with a 26% reintervention rate, underscore the critical necessity of lifelong clinical and radiological surveillance. As established by Schanzer et al. [16], the behavior of the aneurysm sac and the management of intra-graft leaks are paramount to prevent late complications. Our data confirm that mid-to-long-term patient safety after EVAR is fundamentally centered on vigilant, lifelong follow-up, independent of preoperative variables [17,18]. Furthermore, while our findings—and those of similar single-arm registries—are highly encouraging, the current literature is limited by a distinct lack of direct, head-to-head comparisons among contemporary low-profile endografts. Therefore, future prospective, comparative studies are warranted to definitively establish the relative long-term durability and anatomical applicability of these competing systems.

### Study Limitations

Several limitations of the present study should be acknowledged. First, the study design is a single-arm, observational, post-market clinical follow-up without a direct randomized control group (such as open surgical repair or alternative EVAR devices). Second, while the mean follow-up of 2.26 years provides robust data on mid-term primary patency and early endoleak management, longer-term surveillance (e.g., 5 to 10 years) is necessary to fully establish the ultimate durability and late complication rates of the Ankura™ system. Finally, the study cohort was predominantly male (97%). Although this gender distribution accurately reflects the well-established epidemiological prevalence of AAAs, it may limit the generalizability of these findings regarding device performance in female patients, who often present with narrower access vessels and more challenging anatomical constraints. Despite these limitations, the real-world, multicenter nature of this study provides valuable and highly applicable mid-term clinical insights.

## 5. Conclusions

The 97% primary patency rate achieved over a mean mid-term follow-up of 2.26 years demonstrates the ability of next-generation, low-profile endografts, such as the Ankura™ system, to effectively navigate and mitigate challenging anatomical barriers. However, the 26% reintervention requirement—driven primarily by distal disease progression and Type Ib endoleaks rather than structural device failure—highlights that advanced stent-graft technology cannot entirely eliminate the natural morphological evolution of the iliac arteries. Ultimately, the Ankura™ AAA Stent Graft System presents a highly favorable safety and performance profile in a real-world setting. Yet, these outcomes mandate a rigorous, lifelong radiological surveillance strategy to promptly manage post-procedural morphological changes and ensure sustained patient safety.

## Figures and Tables

**Table 1:** Comparison of Demographic, Clinical, and Procedural Characteristics Based on Reintervention Requirement.

Variable	Group	Overall		No Reintervention (*n* = 74)		Reintervention (*n* = 26)		
		*n*	%	*n*	%	*n*	%	*p*-Value
Age	<70 years	29	29.0	23	31.1	6	23.1	0.601
	≥70 years	71	71.0	51	68.9	20	76.9	
Gender	Female	3	3.0	3	4.1	0	0.0	0.708
	Male	97	97.0	71	95.9	26	100.0	
Smoking	No	11	11.0	7	9.5	4	15.4	0.641
	Yes	89	89.0	67	90.5	22	84.6	
Diabetes Mellitus	No	80	80.0	60	81.1	20	76.9	0.864
	Yes	20	20.0	14	18.9	6	23.1	
Coronary Artery Disease	No	64	64.0	47	63.5	17	65.4	1.000
	Yes	36	36.0	27	36.5	9	34.6	
Hypertension	No	10	10.0	9	12.2	1	3.8	0.403
	Yes	90	90.0	65	87.8	25	96.2	
Dyslipidemia	No	49	49.0	37	50.0	12	46.2	0.913
	Yes	51	51.0	37	50.0	14	53.8	
Peripheral Artery Disease	No	86	86.0	64	86.5	22	84.6	1.000
	Yes	14	14.0	10	13.5	4	15.4	
Prior Surgery History	No	65	65.0	48	64.9	17	65.4	1.000
	Yes	35	35.0	26	35.1	9	34.6	
NYHA Classification	NYHA I	49	49.0	38	51.4	11	42.3	0.572
	NYHA II	51	51.0	36	48.6	15	57.7	
ASA Classification	ASA I	3	3.0	1	1.4	2	7.7	0.254
	ASA II	67	67.0	51	68.9	16	61.5	
	ASA III	30	30.0	22	29.7	8	30.8	
Thrombosis	No	97	97.0	71	95.9	26	100.0	0.708
	Yes	3	3.0	3	4.1	0	0.0	
Mortality	Alive	88	88.0	66	89.2	22	84.6	0.790
	Death	12	12.0	8	10.8	4	15.4	
Presence of Endoleak	No	73	73.0	73	98.6	0	0.0	**<0.001**
	Yes	27	27.0	1	1.4	26	100.0	
		**Mean ± SE** **Med (Min**-**Max)**		**Mean ± SE** **Med (Min**-**Max)**		**Mean ± SE** **Med (Min**-**Max)**		***p*** **-value**
Age (years) ^t^		72.01 ± 5.32 72 (51–83)		71.62 ± 5.28 72 (51–83)		73.12 ± 5.37 72.5 (63–83)		0.220
BMI (kg/m^2^) ^t^		25.97 ± 3.43 25.35 (20.32–37.78)		25.67 ± 3.23 25.26 (20.32–37.78)		26.82 ± 3.88 26.2 (20.76–37.4)		0.143
Operative Time (min) ^t^		103.38 ± 25.93 100 (40–160)		104.70 ± 25.88 100 (55–155)		99.62 ± 26.22 100 (40–160)		0.392
Fluoroscopy Time (min) ^z^		16.64 ± 10.05 14 (5–57)		16.85 ± 10.28 14 (5–57)		16.04 ± 9.55 13 (7–50)		0.841
Contrast Amount (mL) ^z^		57.15 ± 25.19 45 (10–150)		58.11 ± 26.19 47.5 (10–150)		54.42 ± 22.33 42.5 (30–100)		0.512
Length of Stay (days) ^t^		3.35 ± 1.30 3 (2–8)		3.38 ± 1.33 3 (2–8)		3.27 ± 1.22 3 (2–8)		0.714
Follow-up Time (years) ^t^		2.26 ± 0.53 2.26 (0.02–3.02)		2.20 ± 0.58 2.25 (0.02–3.02)		2.43 ± 0.34 2.42 (1.96–2.96)		0.056

Statistical significance was defined as *p* < 0.05. Continuous variables are presented as Mean ± SE and Median (Min-Max); comparisons between groups were performed using the independent samples *t*-test (^t^) and Mann–Whitney U Testi (^z^). SE: Standard Error; Med.: Median; Min.: Minimum; Max.: Maximum; BMI: Body Mass Index; NYHA: New York Heart Association; ASA: American Society of Anesthesiologists.

**Table 2:** Characteristics, Timing, and Management Strategies of Endoleaks During Follow-up.

Endoleak Type	Number of Patients (*n* = 27)	Median Time-to-Detection	Management Strategy	Reintervention Modality
Type Ia	3 (11.1%)	3 months	Early Reintervention	Proximal Cuff Placement
Type Ib	23 (85.2%)	12 and 24 months	Elective Reintervention	Distal Extension Deployment
Type II	1 (3.7%)	12 months	Conservative/Surveillance	None (No sac expansion > 5 mm)

**Table 3:** Comparison of Hemodynamic, Laboratory, and Anatomical Measurements Based on Reintervention Requirement.

Variables	Overall	No Reintervention (*n* = 74)	Reintervention (*n* = 26)	
	Mean ± SE Med. (Min.–Max.)	Mean ± SE Med. (Min.–Max.)	Mean ± SE Med. (Min.–Max.)	*p*-Value
Systolic blood pressure(mmHg) ^t^	130.37 ± 8.39 130 (103–150)	130.24 ± 8.76 130 (103–150)	130.73 ± 7.39 130.5 (120–144)	0.800
Diastolic blood pressure (mmHg) ^t^	81.01 ± 7.41 81 (57–93)	80.26 ± 7.94 81 (57–93)	83.15 ± 5.22 82 (71–92)	0.087
Heart Rate (bpm) ^t^	80.18 ± 7.22 79 (65–102)	80.32 ± 7.31 79 (65–101)	79.77 ± 7.09 78.5 (68–102)	0.738
Preoperative Creatinine (mg/dL) ^t^	1.04 ± 0.24 1.00 (0.65–2.07)	1.03 ± 0.26 1.00 (0.65–2.07)	1.05 ± 0.16 1.05 (0.79–1.40)	0.665
Preoperative Blood Urea Nitrogen (BUN) (mg/dL) ^t^	37.79 ± 10.39 39 (10–60)	38.00 ± 11.06 39.5 (10–60)	37.19 ± 8.32 38 (19–54)	0.735
AAA Diameter (mm) ^t^	62.72 ± 10.37 60 (48–100)	62.46 ± 9.24 61 (48–100)	63.46 ± 13.24 57 (50–97)	0.674
Proximal Neck Length (mm) ^z^	20.42 ± 6.12 20 (15–50)	20.97 ± 6.73 20 (15–50)	18.85 ± 3.57 18.5 (15–30)	0.128
Proximal Infrarenal Neck Diameter (mm) ^t^	22.41 ± 3.34 22 (17–30)	22.32 ± 3.20 22 (17–30)	22.65 ± 3.75 21 (17–30)	0.667
Distal Infrarenal Neck Diameter (mm) ^t^	23.19 ± 3.43 23 (18–30)	23.22 ± 3.33 23 (18–30)	23.12 ± 3.78 22.5 (18–30)	0.898
Aortic Neck Angulation (°) ^t^	20.83 ± 15.42 15 (5–57)	21.41 ± 15.69 17.5 (5–57)	19.19 ± 14.83 12.5 (5–50)	0.532
Left Distal Landing Zone Diameter (mm) ^t^	13.49 ± 3.24 13 (9–20)	13.55 ± 3.21 14 (9–20)	13.31 ± 3.37 13 (9–19)	0.740
Right Distal Landing Zone Diameter (mm) ^t^	12.91 ± 3.11 13 (8–20)	13.05 ± 3.02 13 (8–20)	12.50 ± 3.41 12.5 (8–19)	0.438
Left Common Iliac Length (mm) ^t^	56.59 ± 15.07 55.5 (25–100)	57.66 ± 15.61 58 (25–100)	53.54 ± 13.24 52 (28–84)	0.232
Right Common Iliac Length (mm) ^t^	55.48 ± 14.86 55 (25–90)	55.97 ± 14.92 55 (25–90)	54.08 ± 14.88 55 (26–90)	0.578
Infrarenal Aortic Bifurcation Distance (mm) ^z^	117.22 ± 17.94 118 (25–160)	117.19 ± 19.13 119 (25–160)	117.31 ± 14.34 118 (87–150)	0.977
Aortic Bifurcation Diameter (mm) ^t^	34.98 ± 8.67 32.5 (18–70)	34.69 ± 7.93 33 (18–65)	35.81 ± 10.63 30.5 (24–70)	0.574

Statistical significance was set at *p* < 0.05. Continuous variables are presented as Mean ± SE and Median (Min–Max); group comparisons were performed using the independent samples *t*-test (^t^) and Mann–Whitney U Testi (^z^). AAA: Abdominal Aortic Aneurysm; bpm: beats per minute; SE: Standard Error.

**Table 4:** Univariate Logistic Regression Analysis of Factors Associated with Reintervention Requirement in Patients Undergoing Endovascular Abdominal Aortic Aneurysm Repair (EVAR).

Variables	B	SE	*p*-Value	OR (Exp(B))	95% CI
Age ≥ 70 years	0.408	0.529	0.441	1.503	0.533–4.239
Diabetes Mellitus	0.251	0.552	0.649	1.286	0.436–3.794
Coronary Artery Disease	−0.082	0.478	0.864	0.922	0.361–2.351
Hypertension	1.242	1.080	0.250	3.462	0.417–28.748
Peripheral Artery Disease	0.152	0.641	0.813	1.164	0.331–4.089
Preoperative Creatinine	0.412	0.941	0.662	1.510	0.239–9.550
Preoperative BUN	−0.008	0.022	0.732	0.992	0.950–1.036
Abdominal Aortic Aneurysm Diameter	0.009	0.021	0.671	1.009	0.968–1.053
Proximal Neck Length	−0.081	0.054	0.135	0.922	0.830–1.026
Proximal Infrarenal Neck Diameter	0.030	0.068	0.664	1.030	0.902–1.177
Distal Infrarenal Neck Diameter	−0.009	0.067	0.897	0.991	0.870–1.130
Operative Time	−0.008	0.009	0.389	0.992	0.975–1.010
Fluoroscopy Time	−0.008	0.024	0.722	0.992	0.947–1.039
Contrast Amount	−0.006	0.009	0.520	0.994	0.976–1.013

Reintervention requirement was analyzed as the dependent variable and coded as 0 = No and 1 = Yes. Data are presented as the logistic regression coefficient (B), standard error (SE), odds ratio (OR = Exp(B)), and 95% confidence interval (CI). Statistical significance was defined as *p* < 0.05.

## Data Availability

The original contributions presented in this study are included in this article; further inquiries can be directed to the corresponding authors.
